# High-frequency induction heat sintering of 316L stainless steel: a systematic investigation of ultra-low-pressure processing, microstructure, and corrosion performance

**DOI:** 10.1038/s41598-026-65428-1

**Published:** 2026-08-05

**Authors:** Ayman Mahmoud Fahim, Tamás Mikó, Hany S. Abdo, Ubair Abdus Samad, Ibrahim A. Alnaser, Zoltán Gácsi

**Affiliations:** 1 Institute of Physical Metallurgy, Metalforming and Nanotechnology, University of Miskolc, Miskolc, Hungary; 2 Polymer Laboratory, Physics Department, Faculty of Science, Mansoura University, Mansoura, 35516 Egypt; 3 Center of Excellence for Research in Engineering Materials (CEREM), Deanship of Scientific Research, King Saud University, Riyadh, 11421 Saudi Arabia

**Keywords:** High-frequency heat sintering, 316L stainless steel, Ultra-low-pressure processing, Electromagnetic field-enhanced sintering, Powder metallurgy, Vickers hardness, Engineering, Materials science

## Abstract

The present work provides a systematic evaluation of direct pressure-assisted High-Frequency Induction Heat Sintering (HFIHS) of gas-atomized 316 L stainless steel powder at 40 MPa, approximately 50 kHz, 1000–1150 °C, a 5 min hold, and approximately 1 × 10⁻³ Torr. Relative density, Vickers hardness, maximum compressive stress, microstructure, and corrosion behavior in 3.5 wt% NaCl were evaluated. Relative density increased from 88.87% at 1000 °C to 98.61% at 1150 °C. Hardness measurements increased from 156.5 ± 5.2 HV10 to 263.0 ± 14.4 HV10, while the measured maximum compressive stress increased from 284 to 379 MPa. Fiji-based image analysis showed decreasing pore-area fraction, pore size, and pore connectivity, together with increasing pore circularity and grain size. The lowest polarization-derived corrosion rate was obtained at 1100 °C, whereas the 1150 °C specimen had the highest density and a comparatively clean post-corrosion surface. Within the investigated conditions, the results indicate that direct HFIHS can consolidate 316 L stainless steel to near-full density within a short processing cycle and at substantially lower applied pressure than the conventional press-and-sinter baseline used for comparison.

## Introduction

Powder metallurgy (PM) is widely used for stainless-steel components because it supports near-net-shape production, high material utilization, and reduced machining waste^1^. Austenitic 316 L stainless steel is used in structural, chemical, and biomedical environments because of its formability, toughness, and corrosion resistance, which is associated with a Cr-rich passive film and the beneficial effect of Mo on localized corrosion resistance in chloride-containing media^2^.

Conventional press-and-sinter processing of 316 L generally requires high green density, high cold-compaction pressure, and extended furnace exposure to approach full density^3^. Residual porosity and heterogeneous interparticle bonding can reduce mechanical performance and promote occluded electrochemical conditions and pit initiation at pore openings^4,5^. Alternative rapid-consolidation routes include microwave sintering, spark plasma sintering (SPS), and field-assisted sintering technology (FAST), which can shorten processing time but differ in their heating and current-transfer mechanisms^6–8^.

Direct HFIHS uses a high-frequency induction field to heat a conductive die and compact while uniaxial pressure is applied simultaneously. In contrast, indirect induction sintering transfers heat from an inductively heated susceptor or crucible without necessarily applying pressure to the compact, as demonstrated for material-extrusion 316 L parts^9^. SPS is commonly implemented within the broader FAST family and uses low-voltage pulsed or direct current together with uniaxial pressure; the current may pass through the tooling and, depending on electrical conductivity, through the specimen^7,10,11^. Microwave sintering relies on microwave–material or hybrid-heating interactions rather than induction heating^8^. Conventional press-and-sinter processing separates cold compaction from subsequent furnace heating. These distinctions are important because the thermal gradients, current paths, pressure schedules, and cycle times differ among the methods.

Previous work has established rapid HFIHS consolidation for several material classes, including hard metals, high-entropy alloys, aluminium-based composites, iron-based materials, and high-speed steels^12–16^. Related studies have also examined indirect induction sintering of 316 L^9^ and SPS/FAST processing of 316 L^10,11,17^. However, the combined influence of direct pressure-assisted HFIHS processing parameters on the densification, mechanical properties, quantitative microstructural evolution, and electrochemical corrosion behaviour of gas-atomized 316 L stainless steel powder remains insufficiently understood. The present study addresses this gap through a systematic investigation of these interrelated responses under controlled HFIHS processing conditions.

To bridge the gap in rapid powder consolidation literature, this study systematically investigates the direct pressure-assisted HFIHS of gas-atomized 316 L stainless steel powder. Sintering was executed under a unique combination of ultra-low uniaxial pressure (40 MPa) and a high-frequency (~ 50 kHz) electromagnetic field, operating between 1000 °C and 1150 °C with a brief 5-minute isothermal hold under vacuum (~ 1 × 10⁻³ Torr). The primary objective is to elucidate the coupled effects of induction heating and low pressure on densification and microstructural evolution. Furthermore, these structural changes are directly correlated with the resulting mechanical properties (hardness and compressive strength) and electrochemical corrosion behavior in a 3.5 wt% NaCl environment. To highlight the efficiency of this approach, the process window is benchmarked against a previously reported high-pressure press-and-sinter condition (1150 °C at 1600 MPa)^3^, demonstrating a significant advancement in low-pressure manufacturing.

## Experimental procedure

**Fig. 1:**
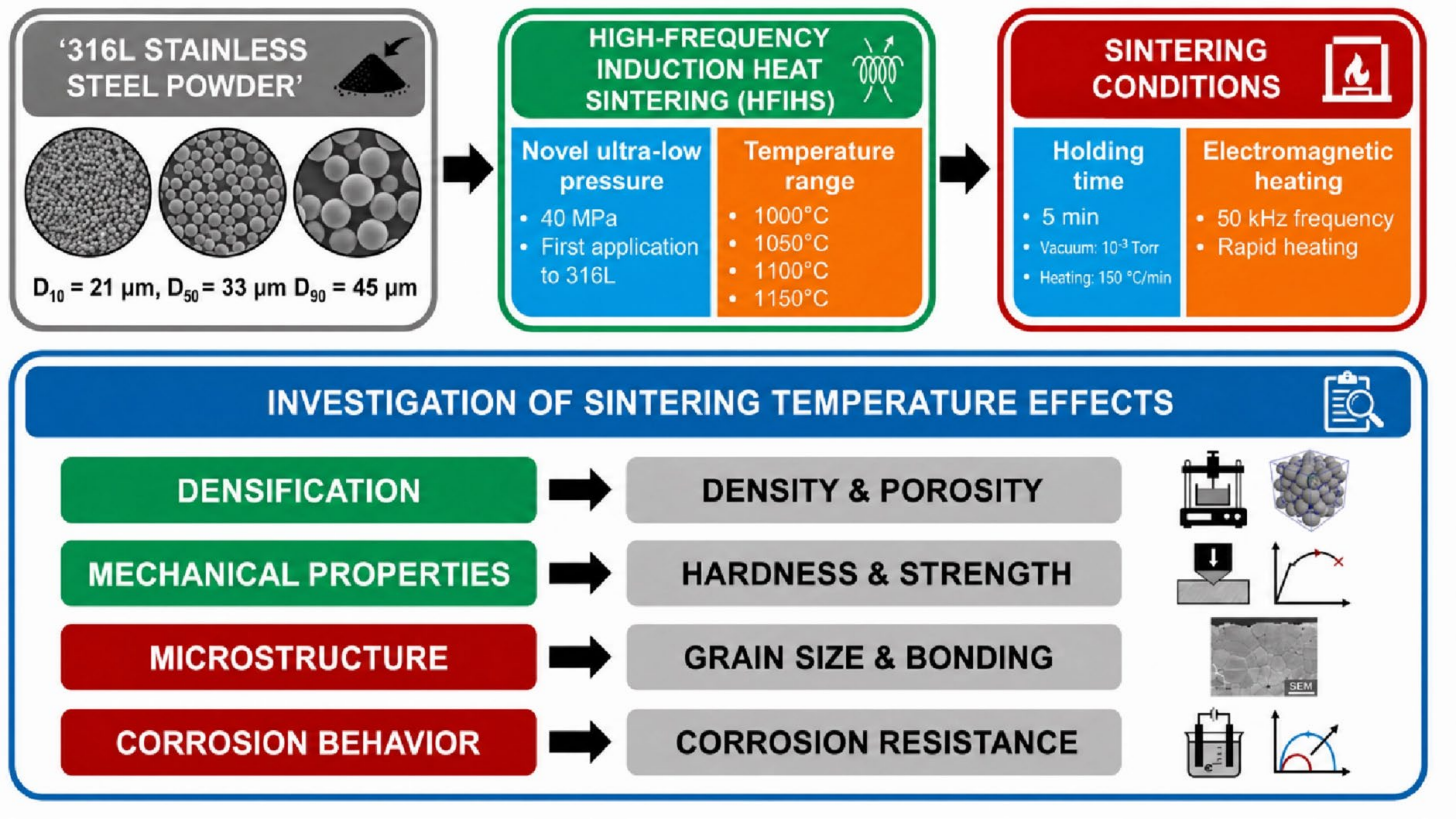
Schematic illustration of the HFIHS processing route and characterization workflow.

Figure 1 summarizes the HFIHS processing route and characterization workflow. Gas-atomized 316 L powder with D₁₀ = 21 μm, D₅₀ = 33 μm, and D₉₀ = 45 μm was consolidated at 40 MPa and approximately 50 kHz. Four target temperatures (1000, 1050, 1100, and 1150 °C) were investigated using a heating rate of 150 °C min⁻¹, a 5 min hold, and a vacuum of approximately 1 × 10⁻³ Torr. The resulting specimens were evaluated for densification, mechanical response, microstructure, and corrosion behavior.

### Initial material characteristics

**Fig. 2:**
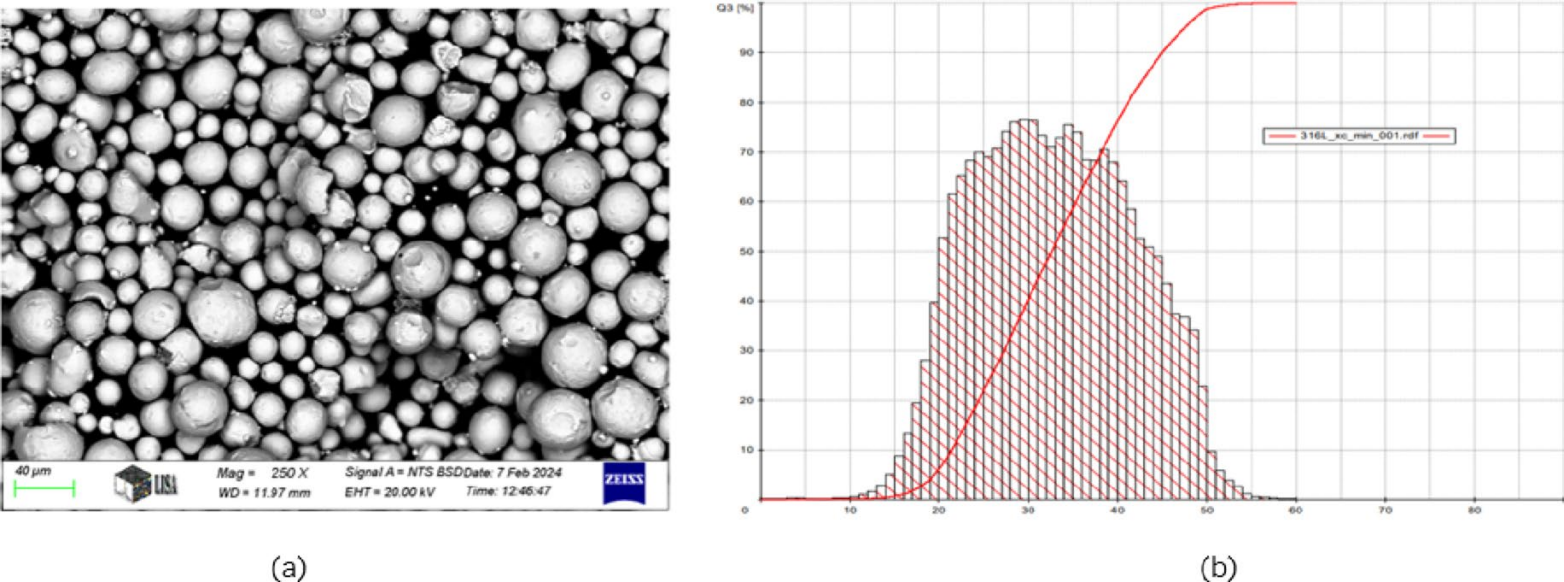
(**a**) Scanning electron microscopy (SEM) image of gas atomized 316 L stainless steel powder. (**b**) Particle size distribution centered at D₅₀ = 33 μm^3^.

Powder characterization was performed at the Institute of Physical Metallurgy, Metalforming and Nanotechnology, University of Miskolc, Hungary. Commercial gas atomized 316 L stainless steel powder (supplied by Oerlikon, USA) was used. The particle size distribution values were as follows: D₁₀ = 21 μm, D₅₀ = 33 μm and D₉₀ = 45 μm. Scanning electron microscopy (SEM) was used to evaluate the morphology of the powder. The chemical composition (in weight%, %) of the powder used in experiments is given in Table 1, as measured by energy dispersive spectroscopy (EDS). As shown in Fig. 2, the particles are predominantly spherical, which is typical of atomized gas powders, although some irregularly shaped particles are also present due to the nature of the atomization process^3^. The particle size distribution exhibits a relatively narrow Gaussian distribution centered around 33 μm.

**Table 1:** Chemical composition of 316 L stainless steel powders based on EDS analysis.

Fe	Cr	Ni	Mo	Si	Mn
64.8 (%)	18.3 (%)	11.6 (%)	2.3(%)	0.7 (%)	2.4 (%)

### High-frequency induction heat sintering (HFIHS)

**Fig. 3:**
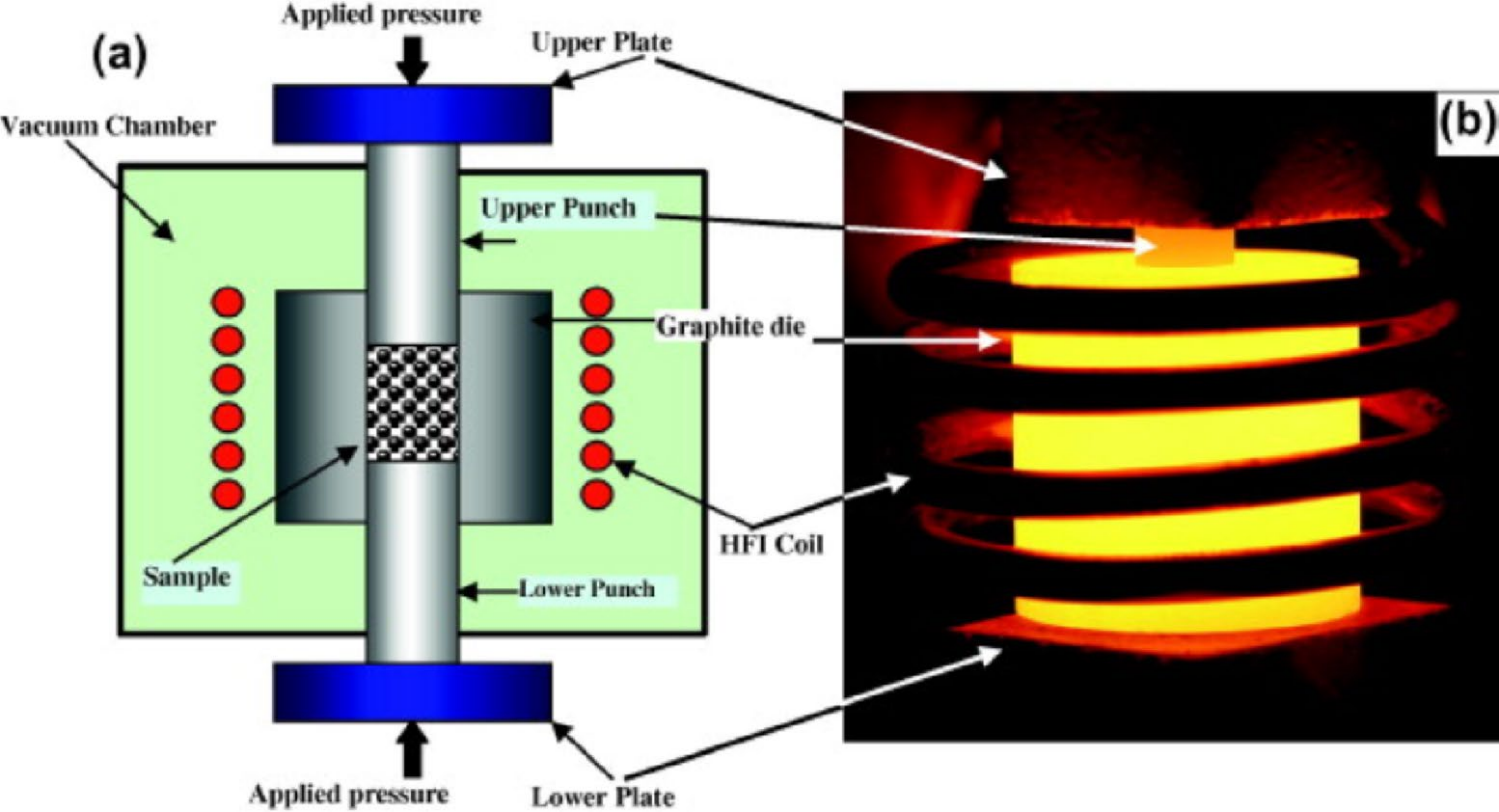
(**a**) Schematic of the High-Frequency Induction Heat Sintering (HFIHS) setup. (**b**) Heating process inside the HFIHS unit during operation^16^.

HFIHS experiments were conducted in the Mechanical Engineering Department, College of Engineering, King Saud University, Riyadh, Saudi Arabia. Figure 3a schematically shows the HFIHS system, and Fig. 3b shows the unit during heating. The setup comprises a uniaxial pressing system, a high-frequency induction heating source, and a vacuum chamber.

Powder was loaded into a graphite die assembly and compressed between upper and lower punches. The die had an outer diameter of 45 mm, an inner diameter of 20 mm, and a height of 40 mm. Before heating, the chamber was evacuated to approximately 1 × 10⁻³ Torr. An induction coil surrounding the die generated an alternating electromagnetic field at approximately 50 kHz, inducing eddy-current heating in the conductive die and powder compact (Fig. 3b). Pressure, displacement, and temperature were monitored during processing. Specimens were heated at 150 °C min⁻¹ to 1000, 1050, 1100, or 1150 °C and held for 5 min. The die-surface temperature was measured with a pyrometer and confirmed with a thermocouple. A pressure of 40 MPa was maintained during heating, holding, and cooling. The specimens were cooled naturally and had final dimensions of approximately 10 mm in diameter and 10–15 mm in height.

### Density and mechanical characterization

Density and mechanical characterization were conducted at the Measurement Laboratory, Mechanical Engineering Department, College of Engineering, King Saud University, Riyadh, Saudi Arabia. A theoretical density of 7.99 g cm⁻³ was used for 316 L stainless steel. Bulk density was measured by the Archimedes method in accordance with ASTM B962-23^18^, and relative density was calculated by normalizing the measured density to the theoretical value. Compression testing was performed at room temperature using an Instron 5982 universal testing machine at a crosshead speed of 3 mm min⁻¹. Cylindrical specimens were approximately 10 mm in diameter with a height-to-diameter ratio of approximately 1.5. Strain was estimated from crosshead displacement, and the reported response is the maximum compressive stress before fracture or macroscopic instability. Vickers hardness was measured using a Wolpert UH930 tester at 10 kgf (HV10) with a 15 s dwell. Six hardness measurements were obtained for each temperature condition and are reported as the mean ± sample standard deviation. Density and maximum compressive stress were each determined from one independent specimen per condition; therefore, these values are reported without error bars and interpreted descriptively.

### Microstructural analysis

Microstructural characterization was performed on all samples to evaluate the effects of sintering temperature and to analyze post-corrosion surface morphology. Optical microscopy and SEM analysis of as-sintered samples were conducted at the Institute of Physical Metallurgy, Metalforming and Nanotechnology, University of Miskolc, Hungary. Post-corrosion SEM/EDS analysis was performed at the SEM Laboratory, Mechanical Engineering Department, College of Engineering, King Saud University, Riyadh, Saudi Arabia.

Polished and etched cross-sections samples sintered at 1000–1150 °C were examined using a Zeiss Axio Imager optical microscope. All optical micrographs were acquired at the same magnification, and included 20 μm scale bar. The as-sintered condition required standard metallographic procedures to prepare samples which included grinding and polishing and chemical etching for grain boundary revelation. The etching solution contained 10 g CuCl₂ and 60 mL distilled water and 80 mL HCl and 50 mL ethanol which required an etching duration of 60 s. The ZEISS EVO 10 MA microscope served to make all necessary observations. The surface morphology of the sample was studied through Secondary electron (SE1) imaging which operated at 500× magnification. The analysis used an accelerating voltage of 20.0 kV and a working distance of 12 mm while the 20 μm scale bar served as a reference for size measurements. The electrochemical tests ended with an evaluation of corroded sample surfaces to detect corrosion-related characteristics. The analysis required the samples to receive a gentle water rinse with distilled water followed by air drying. The FE-SEM (JEOL JSM-7600 F) with EDS system performed characterization of the samples. The samples received a platinum coating which reached a thickness of 25 nm to reduce surface charging effects. The imaging process used secondary electrons at 15 kV accelerating voltage and 4.5 mm working distance. The analysis helped scientists detect specific attack areas and pit formation and surface deterioration events which they verified through microscopic examination of the material structure.

All quantitative image measurements were performed on optical micrographs acquired using an AXIO Imager and processed with Fiji software. Three separate microscope image areas from the polished cross-section were analysed for each sintering temperature of 1000, 1050, 1100, and 1150 °C. The images were calibrated using the embedded 20 μm scale bar. The measured parameters were pore-area fraction, equivalent pore diameter, pore-size distribution, pore circularity, pore number density, a two-dimensional connectivity, and grain size. Pore-area fraction was calculated as the segmented pore area divided by the analysed field area. Equivalent pore diameter was calculated as dₑq = 2(A/π)⁰·⁵, where A is the projected pore area, and pore circularity was calculated as C = 4πA/P², where P is the pore perimeter. Pore number density was calculated as the number of retained pore objects per analysed area. The two-dimensional connectivity was defined as the area of the largest connected pore object divided by the total analysed field area. Grain size was determined from calibrated etched optical micrographs using a Fiji/ImageJ-based equivalent image-analysis method, and the values for each condition are reported as the arithmetic mean ± standard deviation for each condition.

### Electrochemical corrosion testing

Electrochemical corrosion testing was performed at the Mechanical Engineering Department, College of Engineering, King Saud University, Riyadh, Saudi Arabia. Electrochemical corrosion behavior was evaluated using a three-electrode cell configuration, with the sintered samples serving as the working electrode, a platinum counter electrode, and an Ag/AgCl reference electrode. The measurements were carried out in a 3.5 wt% NaCl aqueous solution at room temperature using an Autolab PGSTAT 30 electrochemical workstation (Metrohm Autolab, Amsterdam, The Netherlands).

Electrochemical impedance spectroscopy (EIS) measurements were performed using a sinusoidal perturbation of ± 5 mV, with frequency scanning range from 100k Hz to 100 mHz. The impedance data were analyzed using NOVA software (version 1.814). Linear polarization resistance (LPR) tests were conducted relative to the open-circuit potential with a scan rate of 5 mV s⁻¹ after 72 h of immersion in the electrolyte.

## Results and discussion

### Densification behavior

**Fig. 4:**
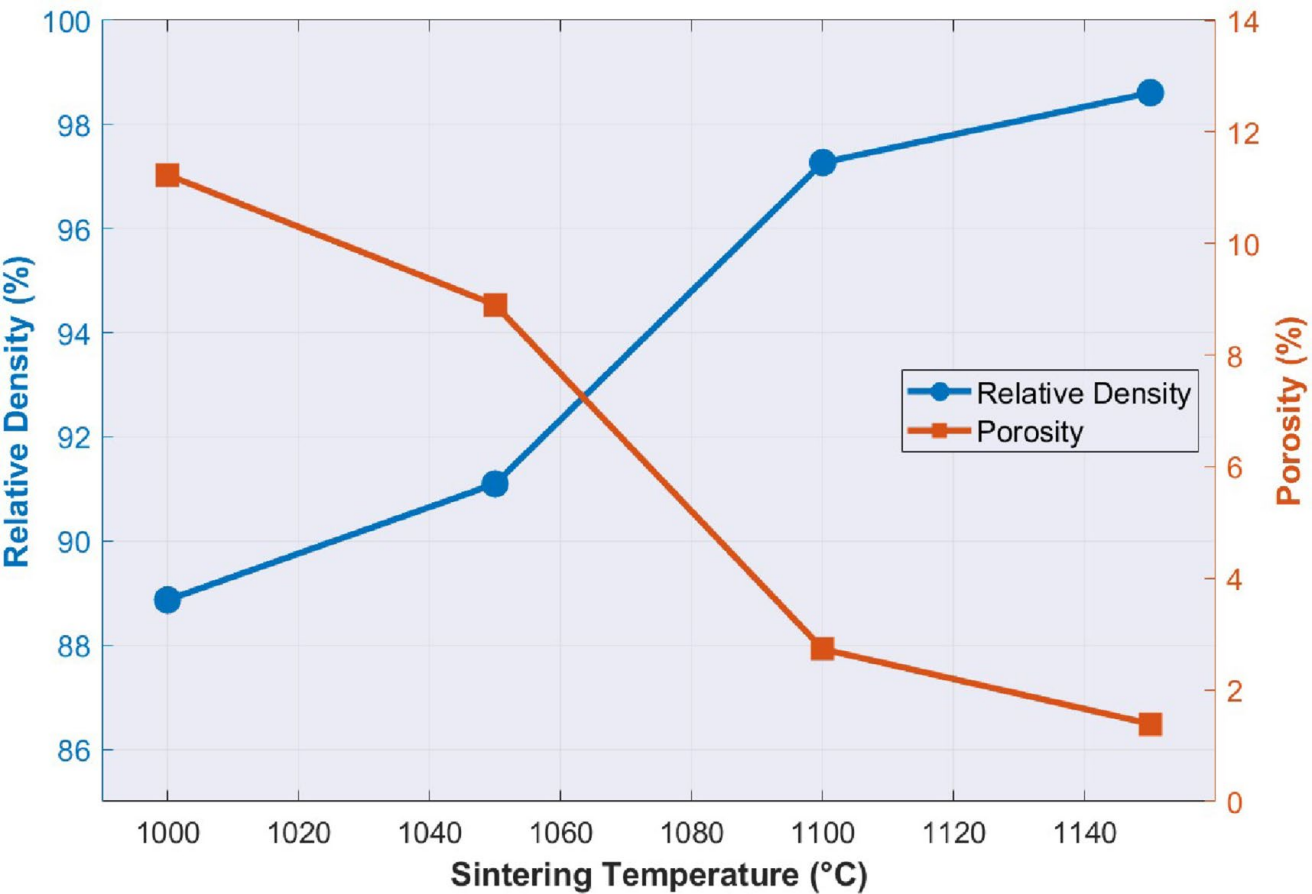
Relative density (left axis) and density-derived porosity (right axis) of HFIHS-processed 316 L stainless steel as functions of sintering temperature.

Figure 4 presents the relative density evolution of 316 L stainless steel as a function of sintering temperature under HFIHS processing at 40 MPa pressure. The results show a well-organized densification pattern which contradicts typical beliefs about how to process powder metallurgy materials. The relative density showed a continuous increase from 88.87% at 1000 °C until it reached 98.61% at 1150 °C which resulted in a 9.74% improvement of densification throughout the studied temperature interval.

Relative density increased from 88.87% at 1000 °C to 91.11% at 1050 °C, 97.27% at 1100 °C, and 98.61% at 1150 °C. The largest increment occurred between 1050 and 1100 °C. This trend is consistent with accelerated neck growth and mass transport over this temperature interval, followed by a smaller densification increment between 1100 and 1150 °C as the residual pore fraction approached a low level.

The rapid heating and simultaneous application of pressure during HFIHS promote particle rearrangement, neck formation, and diffusion, thereby accelerating densification. The combined thermal and mechanical driving forces enhance interparticle bonding and pore shrinkage, enabling the development of a highly consolidated microstructure within a short processing cycle.

The porosity evolution, shown on the secondary axis of Fig. 4, provides complementary insight into the densification mechanism. The porosity decreased from 11.13% at 1000 °C to 1.39% at 1150 °C, representing an 87.5% reduction in void content. This sharp transition indicates the activation of enhanced diffusion mechanisms under electromagnetic field influence, which effectively eliminate interconnected porosity and promote the formation of isolated, spherical pores that are subsequently eliminated at higher temperatures. This trend agrees with the quantitative cross-sectional image analysis, which showed decreasing pore-area fraction, mean pore size, and pore connectivity with increasing temperature. The results are consistent with progressive pore shrinkage, rounding, and isolation during sintering.

At 1150 °C, HFIHS produced a relative density of 98.61% at 40 MPa. Under the conventional press-and-sinter baseline reported previously, 94.62% relative density was obtained at the same sintering temperature after cold compaction at 1600 MPa^3^. This comparison indicates that the investigated HFIHS condition achieved a higher final density while using a lower applied pressure and a shorter hold. Accordingly, comparisons with alternative consolidation routes should be interpreted with caution, as the reported densification response is strongly influenced by powder characteristics, specimen geometry, processing atmosphere, thermal cycle, applied pressure, temperature-monitoring approach, and the method used to determine density^10,11,19,20^.

### Mechanical properties

#### Vickers hardness

**Fig. 5:**
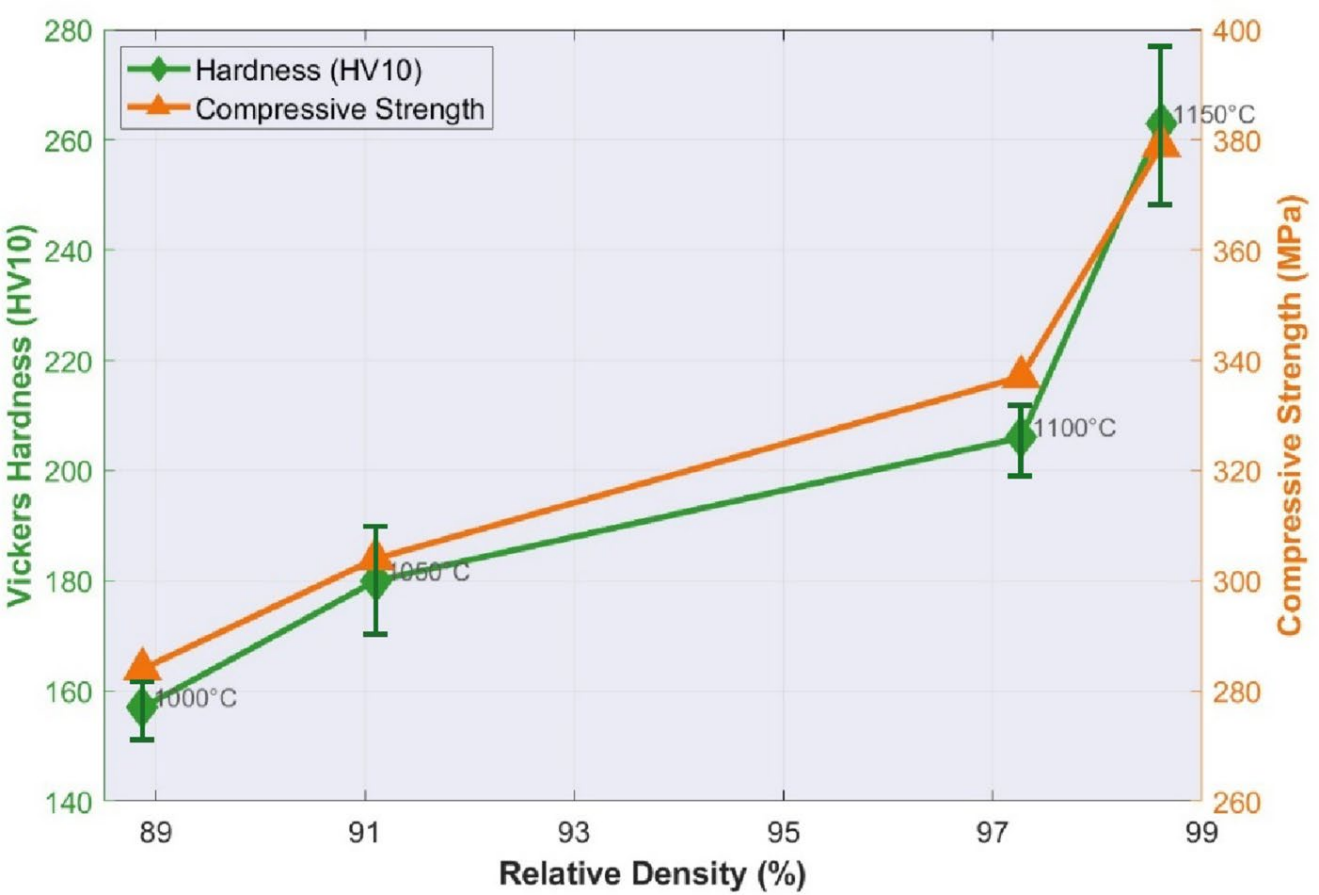
Mean Vickers hardness and maximum compressive stress of HFIHS-processed 316 L stainless steel as functions of relative density.

Figure 5 shows the hardness results with standard-deviation error bars and the condition-level maximum compressive stress values. Mean hardness increased from 156.5 ± 5.2 HV10 at 1000 °C to 180.3 ± 9.9 HV10 at 1050 °C, 205.7 ± 6.4 HV10 at 1100 °C, and 263.0 ± 14.4 HV10 at 1150 °C. The increase from 1000 to 1150 °C was approximately 68%. The corresponding increase in relative density and reduction in pore content indicate that consolidation was the dominant factor associated with the hardness trend.

The hardness of 263.0 ± 14.4 HV10 achieved in this study is particularly noteworthy when compared to other processing methods. The hardness level of furnace-sintered press-and-sinter 316 L materials reaches 150–200 HV based on their density and sintering process parameters^3^. Laser powder bed fusion/SLM processing has been reported to achieve hardness values around 237 HV^21^, while Spark Plasma Sintering (SPS) can reach hardness values up to ~ 300–350 HV in ultrafine-grained 316 L, but often requires powder pre-processing (e.g., intensive ball milling) and more complex processing routes^11^. The 263 HV10 hardness level achieved in this research makes HFIHS an attractive processing technique because it produces better hardness than standard sintering methods while matching SPS results with less complicated and shorter production times.

The hardness increase was associated mainly with improved densification, reduced porosity, stronger interparticle bonding, and a lower defect content. Grain coarsening would normally reduce grain-boundary strengthening; however, within the investigated conditions, this effect was outweighed by the substantial increase in consolidation and effective load-bearing area.

The measured hardness suggests that the HFIHS-processed material may be suitable for applications requiring a combination of moderate hardness and corrosion resistance, including valve seats, pump components, and fluid-handling equipment used in chemical-processing or marine environments^22^. Increased hardness in 316 L stainless steel has also been associated with reduced material loss under sliding wear, indicating potential for wear-exposed components in chemical-processing, food-processing, and biomedical equipment. However, tensile, fatigue, wear, and environment-specific corrosion testing is required before the material can be qualified for these demanding applications.

#### Compressive strength

The compressive strength evolution, shown alongside hardness in Fig. 5, demonstrates a similar strong dependence on relative density. The results show a clear and significant improvement in compressive strength with increasing densification, mirroring the trends observed in hardness. The compressive strength increased systematically from 284 MPa at 88.87% relative density (1000 °C) to 379 MPa at 98.61% relative density (1150 °C), representing a 33% increase across the investigated densification range.

The maximum compressive stress measured at 1150 °C is comparable in magnitude to values reported for some conventionally sintered PM 316 L materials, although direct comparison is limited by differences in specimen geometry, density, strain measurement, and the definition of compressive strength^19,21^.

Compared with more complex processing routes and modified alloys, the present unalloyed 316 L specimens were consolidated without ball-milling pretreatment. The result supports the feasibility of HFIHS for rapid consolidation, but it does not establish equivalence to tensile yield strength or performance under service loading.

The observed increase in maximum compressive stress is consistent with the removal of load-reducing pores, a larger effective load-bearing cross-section, and improved particle bonding. These associations are supported by the density and image-analysis trends, although the relative contributions of grain size, texture, residual stress.

The strong correlation between relative density and mechanical properties, as illustrated in Fig. 5, confirms that densification is the primary mechanism responsible for property enhancement in HFIHS processing. Both hardness and compressive strength exhibit near-linear relationships with density in the lower density range (88–92%), followed by accelerated improvement at higher densities (> 95%). This behavior indicates that the elimination of interconnected porosity and the achievement of near-full densification are critical for unlocking the exceptional mechanical performance demonstrated in this study.

### Microstructural characteristics of as-sintered samples

**Fig. 6:**
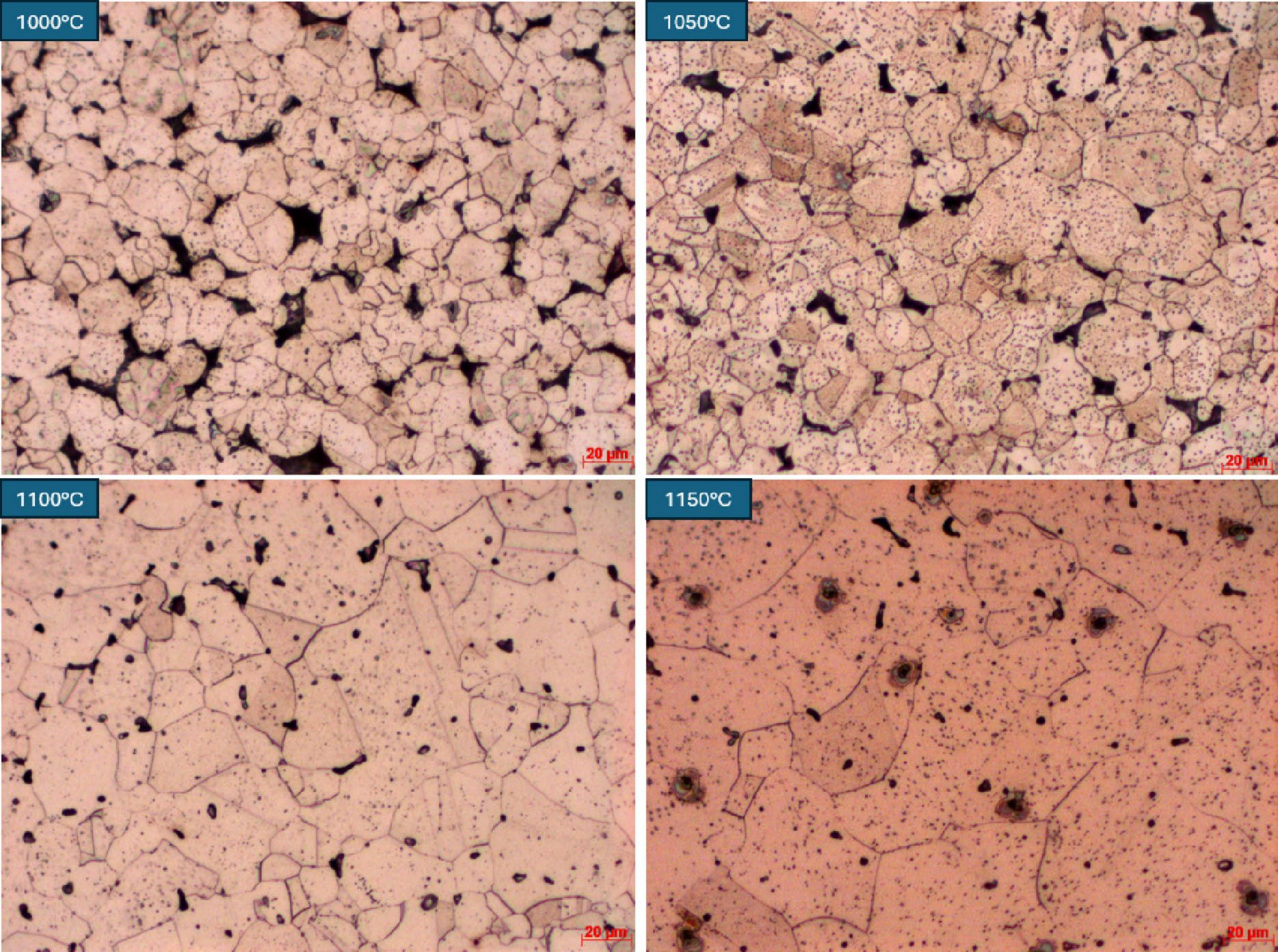
Optical micrographs of polished and etched cross-sections of HFIHS-processed 316 L stainless steel sintered at 1000, 1050, 1100, and 1150 °C under 40 MPa with a 5 min hold.

**Fig. 7:**
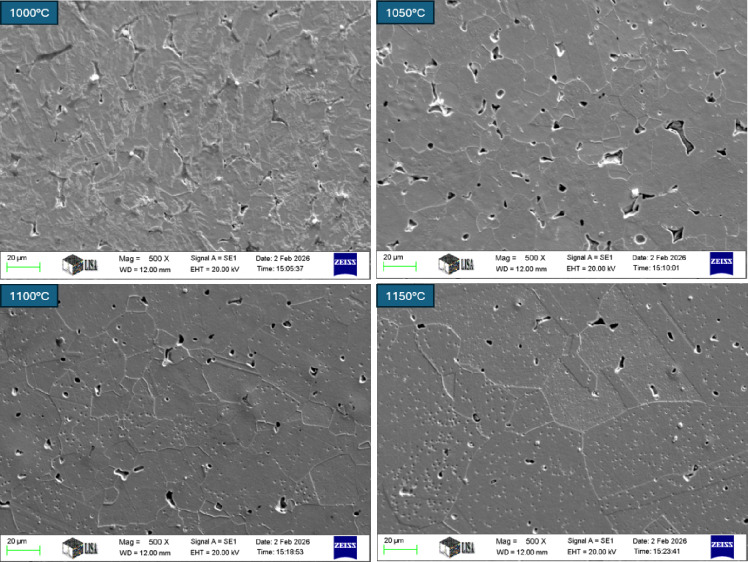
SEM micrographs of etched, as-sintered 316 L stainless steel specimens processed by HFIHS at 1000, 1050, 1100, and 1150 °C under 40 MPa with a 5 min hold.

Optical microscopy (Fig. 6) and SEM (Fig. 7) were used to examine pore morphology, grain structure, and particle bonding in the HFIHS-processed specimens.

#### Porosity evolution and densification behavior

In the optical micrographs, dark pores contrast with the metallic matrix, allowing the evolution of pore size, shape, and distribution with sintering temperature to be compared.

At 1000 °C, the cross-section contained numerous large, irregular pores, including connected pore regions. This morphology is consistent with incomplete interparticle bonding and the lower measured density. Connected pores can reduce the effective load-bearing area and provide pathways for electrolyte ingress.

As the sintering temperature increased to 1050 °C, a noticeable reduction in both the number and size of pores is observed. The pores develop into smaller rounded openings which separate from each other. The sintering process continues because surface tension causes pores to change their shape into spherical forms which represent their most energy-efficient state before they vanish completely. The densification process achieves its highest point when porosity transforms from connected to separate spaces because this transition creates material resistance to fluid penetration and enhances its mechanical properties.

At 1100 °C, the microstructure was substantially denser, with fewer and smaller residual pores than at 1000 and 1050 °C. The relative density reached 97.27%, and quantitative image analysis confirmed a marked reduction in pore-area fraction and connectivity. Residual pores remained and were predominantly small and isolated.

At 1150 °C, the optical fields contained a low area fraction of mostly small, isolated pores rather than a completely pore-free structure. This observation is consistent with the measured relative density of 98.61% and the image-analysis pore-area fraction of 1.38 ± 0.21%. The residual porosity and pronounced grain growth indicate that the microstructure approached, but did not reach, full density.

#### Grain structure development and particle bonding

The SEM micrographs complement the optical analysis by showing the progressive development of particle bonding and grain-boundary definition with increasing sintering temperature. These images are interpreted together with the quantitative Fiji-based grain-size and pore measurements rather than as stand-alone evidence of mechanical performance.

The grain structure shows no clear definition at 1000 °C because the grain boundaries remain difficult to identify. The original powder particles remain visible in certain areas because the necking between particles has not reached full development which shows that particle bonding remains incomplete. The surface shows an uneven, rough texture because it contains multiple defects which create irregular patterns. The material shows no clear grain boundary definition because temperature conditions have not allowed substantial grain growth which maintains its powder-like structure.

At 1050 °C, grain boundaries and interparticle necks were more distinct, and the surface appeared more uniform than at 1000 °C. The fine grain structure and remaining pores indicate that consolidation was still incomplete.

At 1100 °C, grain boundaries were more clearly resolved and the microstructure appeared more consolidated than at 1000 and 1050 °C. Fiji-based analysis gave a mean grain size of 37.74 ± 11.15 μm. The concurrent reduction in pore-area fraction and connectivity is consistent with improved particle bonding and density.

At 1150 °C, larger equiaxed grains were visible, and Fiji-based analysis yielded a mean grain size of 61.90 ± 13.93 μm. The limited additional decrease in pore-area fraction between 1100 and 1150 °C, together with pronounced grain coarsening, suggests a transition from densification-dominated evolution toward grain-growth-dominated evolution.

#### Quantitative microstructural analysis

**Fig. 8:**
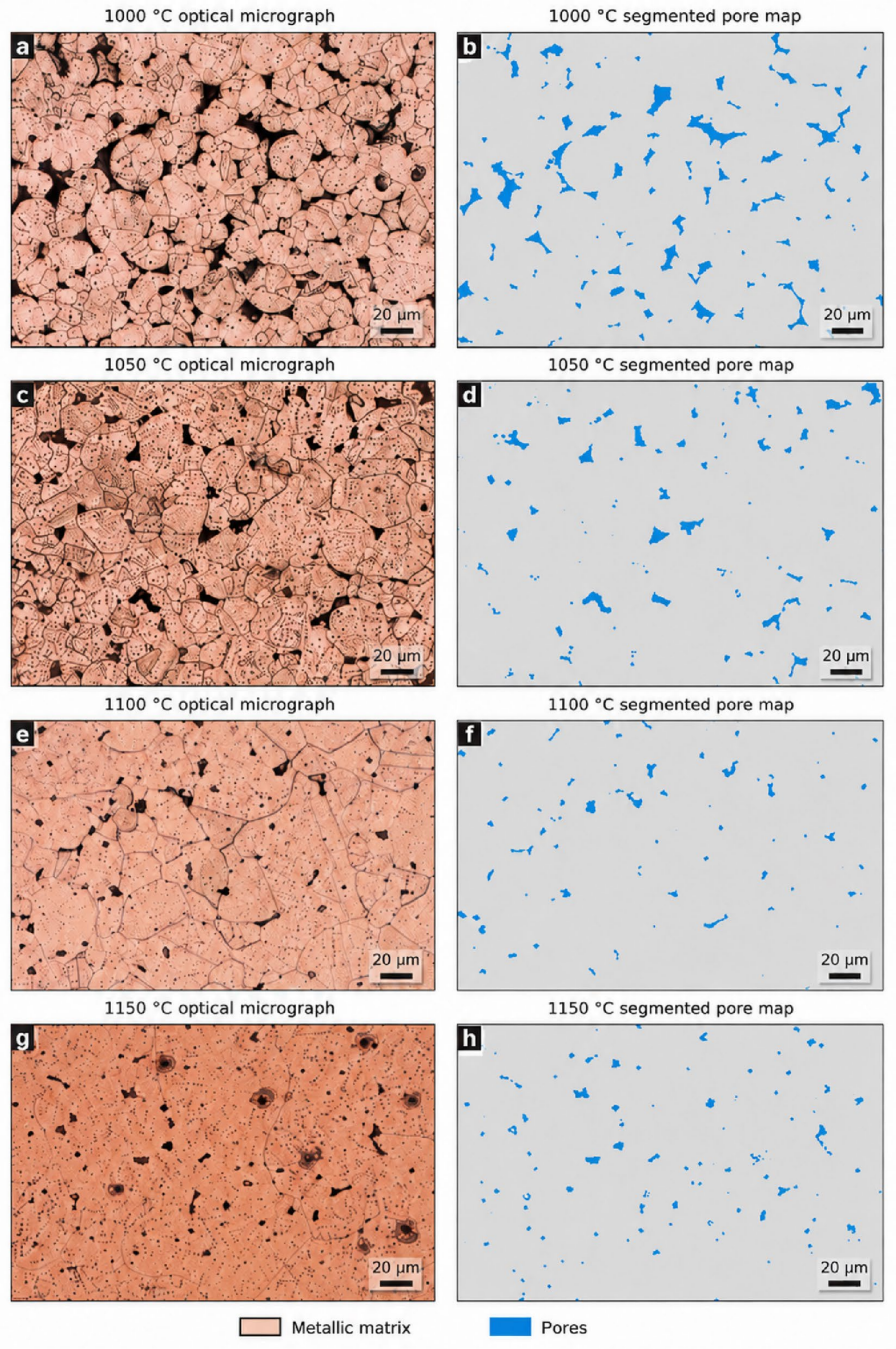
Representative etched optical micrographs and corresponding binary pore-segmentation maps for specimens sintered at 1000, 1050, 1100, and 1150 °C. The same image calibration and analysed area were used for all conditions. Blue regions represent retained pore objects; scale bars: 20 μm.

Figure 8 shows representative optical micrographs and corresponding segmented pore maps. The same calibration and analysis procedure was applied to all four conditions. With increasing temperature, large irregular pore regions decreased and the residual pores became finer and more isolated.

**Fig. 9:**
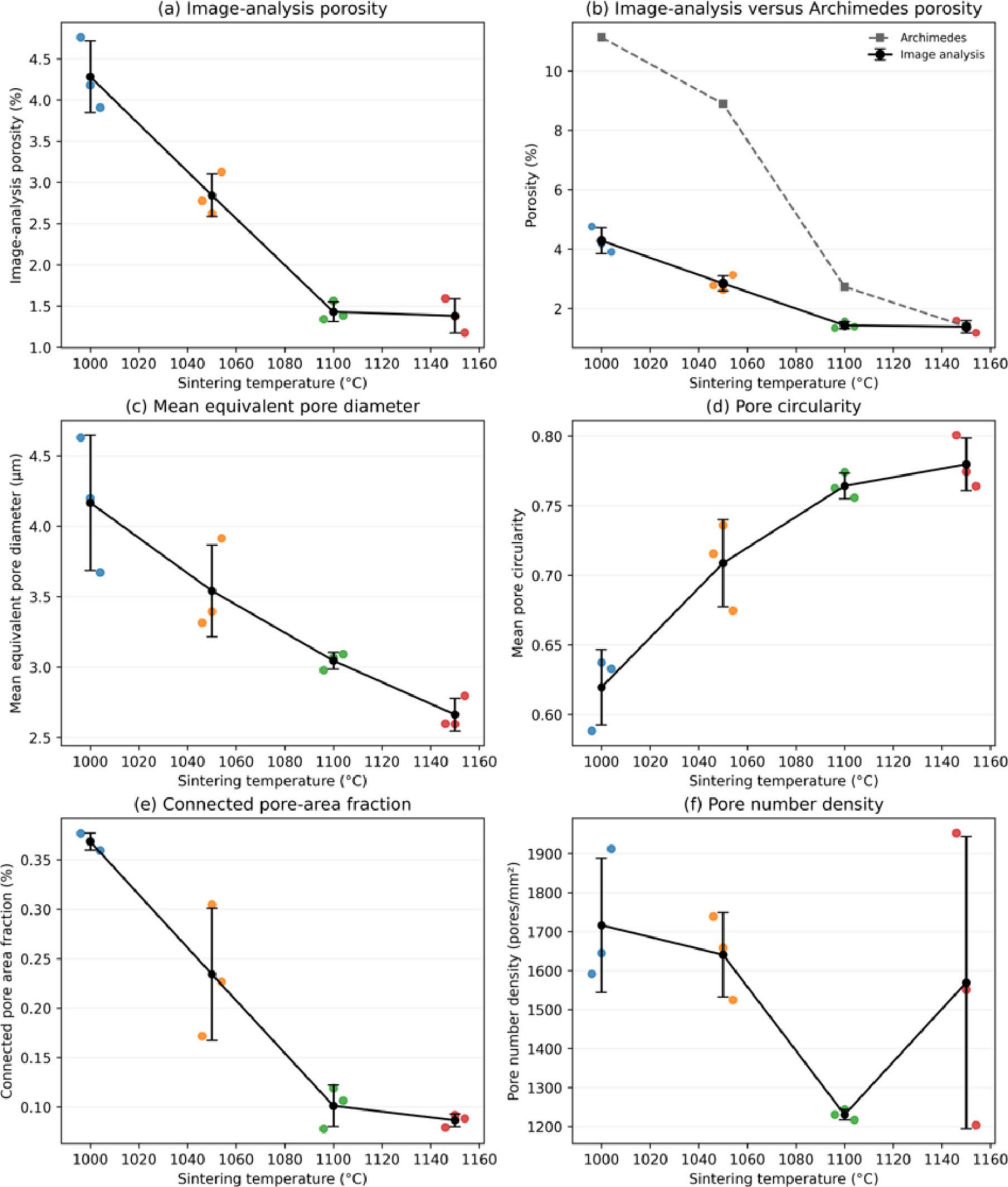
Quantitative evolution of pore characteristics with sintering temperature: (**a**) image-analysis porosity; (**b**) comparison between image-analysis and Archimedes porosity; (**c**) mean equivalent pore diameter; (**d**) mean pore circularity; (**e**) largest connected pore-area fraction, used as a two-dimensional connectivity; and (**f**) pore number density. Colored points represent the three analysed optical fields, black symbols show the field mean, and error bars represent the standard deviation among the three fields.

The quantitative results obtained from three optical fields per condition are summarized in Table 2; Fig. 9. The image-analysis pore-area fraction decreased from 4.28 ± 0.44% at 1000 °C to 2.84 ± 0.26% at 1050 °C, 1.43 ± 0.12% at 1100 °C, and 1.38 ± 0.21% at 1150 °C. The largest decrease occurred between 1050 and 1100 °C, consistent with the pronounced increase in relative density from 91.11% to 97.27% over the same interval. The small additional decrease between 1100 and 1150 °C indicates that most densification had already occurred by 1100 °C.

**Fig. 10:**
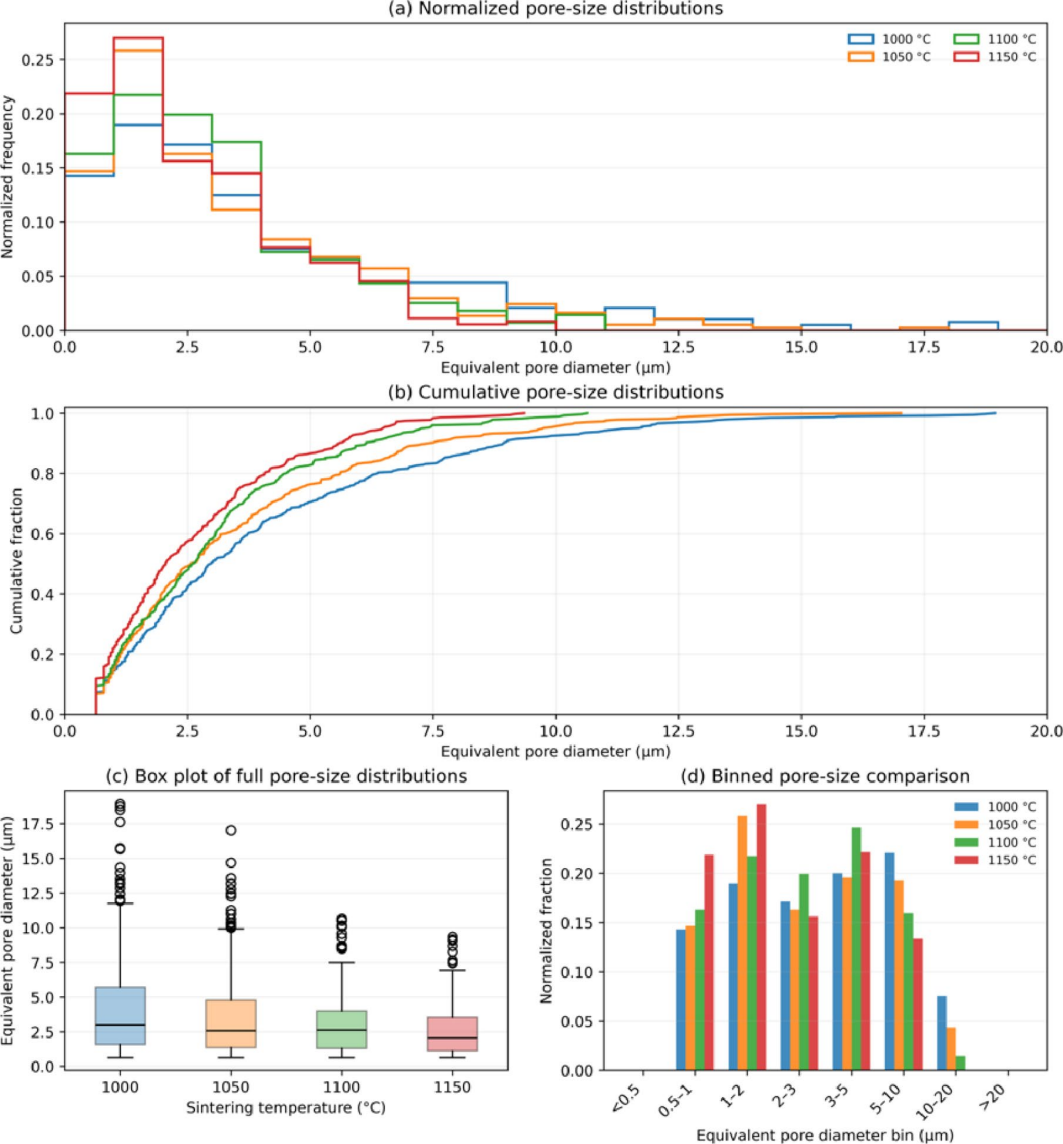
Fiji-derived equivalent pore-size distributions for specimens sintered at 1000, 1050, 1100, and 1150 °C: (**a**) normalized pore-size distributions; (**b**) cumulative distributions; (**c**) box plots showing the median, interquartile range, whiskers, and outliers; and (**d**) normalized fractions in selected pore-size intervals.

Mean equivalent pore diameter decreased from 4.17 ± 0.48 μm at 1000 °C to 3.54 ± 0.33 μm at 1050 °C, 3.05 ± 0.06 μm at 1100 °C, and 2.66 ± 0.12 μm at 1150 °C. The pore-size distributions in Fig. 10 shifted progressively toward smaller diameters, with a reduced large-pore tail, indicating pore shrinkage and increasing isolation of residual interparticle voids.

Mean pore circularity increased from 0.619 ± 0.027 at 1000 °C to 0.709 ± 0.031 at 1050 °C, 0.764 ± 0.009 at 1100 °C, and 0.780 ± 0.019 at 1150 °C. This increase is consistent with progressive pore spheroidisation driven by surface-energy minimization during sintering. In parallel, the largest connected pore-area fraction decreased from 0.368 ± 0.009% at 1000 °C to 0.234 ± 0.067% at 1050 °C, 0.101 ± 0.021% at 1100 °C, and 0.086 ± 0.006% at 1150 °C. The decrease indicates progressive separation and closure of interconnected pore channels rather than only a reduction in total pore area.

Pore number density decreased from 1716 ± 172 mm⁻² at 1000 °C to 1641 ± 108 mm⁻² at 1050 °C and 1230 ± 13 mm⁻² at 1100 °C, before increasing to 1569 ± 375 mm⁻² at 1150 °C. This final increase does not contradict the continued reduction in total pore area and mean pore diameter; instead, it indicates that the residual porosity was distributed among a larger number of smaller isolated pores, with substantial field-to-field variation.

**Table 2:** Quantitative pore characteristics obtained from Fiji optical image analysis.

Sintering temperature (°C)	Image porosity (%)	Mean dₑq (µm)	Circularity	Connected area (%)	Pores per field	Pore density (mm⁻²)
1000	4.28 ± 0.44	4.17 ± 0.48	0.619 ± 0.027	0.368 ± 0.009	128 ± 13	1716 ± 172
1050	2.84 ± 0.26	3.54 ± 0.33	0.709 ± 0.031	0.234 ± 0.067	123 ± 8	1641 ± 108
1100	1.43 ± 0.12	3.05 ± 0.06	0.764 ± 0.009	0.101 ± 0.021	92 ± 1	1230 ± 13
1150	1.38 ± 0.21	2.66 ± 0.12	0.780 ± 0.019	0.086 ± 0.006	117 ± 28	1569 ± 375

Fiji/ImageJ-based grain-size analysis showed that the mean grain size increased from 16.48 ± 3.40 μm at 1000 °C to 24.41 ± 4.52 μm at 1050 °C, 37.74 ± 11.15 μm at 1100 °C, and 61.90 ± 13.93 μm at 1150 °C. The increase reflects enhanced grain-boundary mobility with increasing temperature. As densification progressed and the pore network became smaller and more isolated, the restraint imposed by residual pores decreased, allowing more pronounced grain growth. The large increase at 1150 °C, despite only a small further decrease in pore-area fraction, indicates a transition from densification-dominated evolution toward grain-growth-dominated evolution.

The quantitative microstructural trends are consistent with the measured changes in bulk properties. Reduced pore-area fraction, smaller pore size, lower connectivity, and improved interparticle consolidation increased the effective load-bearing cross-section and reduced defect-related stress concentrations. Accordingly, the hardness increase is attributed mainly to improved densification, reduced porosity, stronger interparticle bonding, and reduced defect content. Grain coarsening would normally reduce grain-boundary strengthening, but this effect was outweighed by the substantial improvement in densification and consolidation. The reduction in connected porosity is also consistent with reduced electrolyte-access pathways and the improved electrochemical response observed from 1000 to 1100 °C.

### Corrosion behavior analysis

#### Electrochemical impedance spectroscopy (EIS)

**Fig. 11:**
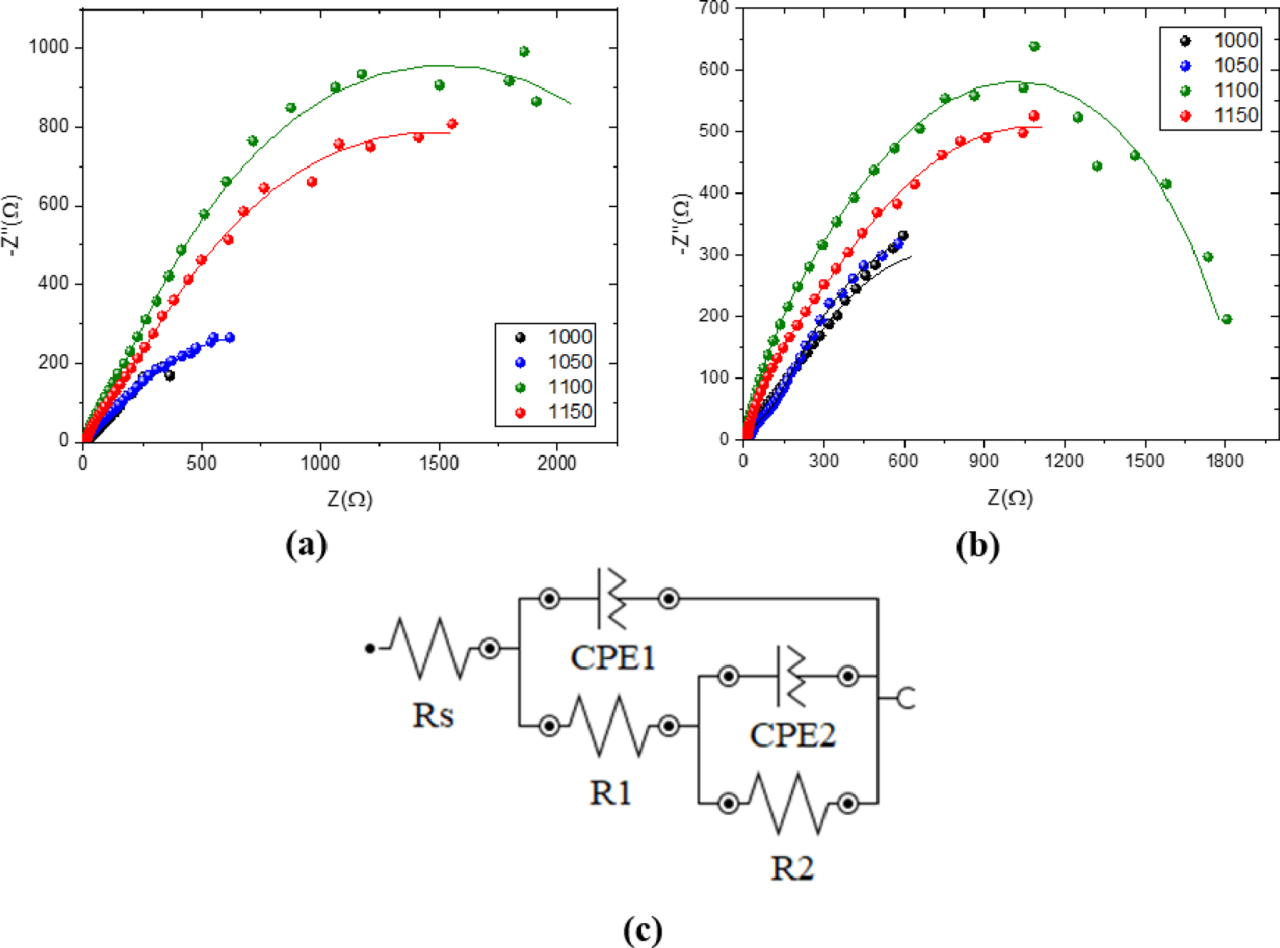
Nyquist plots of HFIHS-processed 316 L stainless steel specimens sintered at 1000, 1050, 1100, and 1150 °C, measured in 3.5 wt% NaCl solution after (**a**) 24 h and (**b**) 72 h exposure, together with (**c**) the equivalent circuit model used for fitting.

The corrosion behavior of the HFIHS-processed 316 L stainless steel samples was investigated using Electrochemical Impedance Spectroscopy (EIS) in a 3.5% NaCl solution. The Nyquist plots, presented in Fig. 11, illustrate the electrochemical response of the samples after 24 and 72 h of exposure. The plot shows a clear trend of increasing semicircle diameter with higher sintering temperatures, indicating enhanced corrosion resistance^23^.

To quantitatively analyze the EIS data, the results were fitted to the equivalent circuit model shown in Fig. 11c. This two-time-constant model (Rs(CPE1-R1)(CPE2-R2)) was selected because it accurately represents the dual-layer electrochemical interface characteristic of porous sintered stainless steel: the first time constant (CPE1-R1) describes the response of the outer corrosion product or oxide layer, while the second time constant (CPE2-R2) captures the charge-transfer process at the underlying metal surface. The quality of fit was confirmed by chi-square values below 10⁻³ for all samples. This model consists of the solution resistance (Rs), a constant phase element (CPE1) and resistance (R1) related to the corrosion product layer, and another constant phase element (CPE2) and resistance (R2) associated with the charge transfer process at the metal-electrolyte interface^23,24^. The physical meaning of the components in the proposed circuit are as follows: R2 and CPE2 represent the resistance and constant phase element (double layer capacitance) associated with the charge transfer process occurring on the metal surface at low frequency; R1 and CPE1 represent the resistance and capacitance of the redox transformation of corrosion products on the surface at moderate frequency; and Rs represents the ohmic resistance of the solution at higher frequencies. The fitted parameters are summarized in Table 3.

**Table 3:** Parameters obtained from fitting circuits after exposure periods.

Sample	Rs (Ω)	CPE-1 (Mho)	n1	R1 (Ω)	CPE-2 (Mho)	n2	R2 (Ω)
1000 (24 h)	15.7	0.00235	0.48	177	0.00351	0.70	671
1050 (24 h)	10.01	0.00080	0.65	91.1	0.00323	0.45	1349
1100 (24 h)	8.86	0.00044	0.74	449	0.00055	0.78	2465
1150 (24 h)	9.66	0.00096	0.67	401	0.00148	0.69	2367
1000 (72 h)	10.7	0.00148	0.57	219	0.00439	0.57	1134
1050 (72 h)	17.8	0.00151	0.58	137	0.00501	0.60	1288
1100 (72 h)	6.65	0.000346	0.75	860	0.00687	0.77	1016
1150 (72 h)	7.52	0.00072	0.70	525	0.00169	0.64	1436

The constant phase element (CPE) values were observed to decrease with increased sintering temperature. This trend can be attributed to the reduction in surface porosity at higher sintering temperatures, resulting in a more compact and homogeneous microstructure compared to samples sintered at lower temperatures. The higher surface heterogeneity of the low-temperature sintered samples is reflected by their comparatively higher CPE values.

After 24 h of exposure, the resistances associated with surface redox processes (R1) and the charge transfer process (R2) were both significantly higher for samples sintered at elevated temperatures. Polarization resistance (R1) is a key parameter for estimating ion release from alloys in the given environment. Higher R1 values indicate reduced ion release and consequently a lower corrosion rate. Similarly, higher R2 reflects enhanced protective ability, reduced ion transfer, and improved corrosion resistance. The increase in R2 with sintering temperature is consistent with a more resistive electrochemical interface; however, it does not by itself establish passive-film composition or structure. Specifically, the R2 value increased from 671 Ω for the 1000 °C sample to 2465 Ω for the 1100 °C sample after 24 h, representing a 267% improvement in charge transfer resistance.

The increased diameter of the capacitive semicircle observed in the Nyquist plots for samples sintered at higher temperatures further confirms the increase in polarization resistance (R1), indicating a more resistive and protective electrochemical interface. Taken together, these electrochemical results show that increasing the sintering temperature improved the impedance response under the test conditions. The specimens sintered at 1100 and 1150 °C exhibited the highest impedance response; this is consistent with the effects of increased densification, although the relative contributions of passive-film characteristics and microstructure were not measured directly.

Upon prolonged exposure of 72 h, the electrochemical response of the low- temperature sintered samples exhibited an increase in CPE1 values, corresponding to the formation of corrosion products associated with surface redox reactions, along with increased CPE2 values related to the charge transfer process at the metal surface. This behavior indicates that samples sintered at lower temperatures are more vulnerable to corrosion due to their less compact and porous surface structure, which facilitates the ingress of aggressive corrosive species. The sustained increase in CPE values upon prolonged exposure suggests that the porous structure of the low- temperature sintered samples continuously expose fresh reactive surfaces beneath the corrosion products, thereby promoting ongoing electrochemical activity which results in accelerated corrosion.

In contrast, samples sintered at higher temperatures exhibited consistently lower CPE1 and CPE2 values and higher resistance values (R1 and R2) even after 72 h of exposure. This behavior is consistent with a more resistive interfacial response that limits charge transfer and electrolyte access; however, it does not directly establish the formation, composition, or long-term stability of a passive layer. The high resistance values indicate that the higher-temperature specimens maintained a more protective electrochemical response during the 72 h exposure. Overall, the equivalent-circuit results suggest an association between higher sintering temperature, surface compactness, and improved corrosion resistance, but direct surface-chemistry measurements are required to evaluate passive-layer integrity.

#### Potentiodynamic polarization

**Fig. 12:**
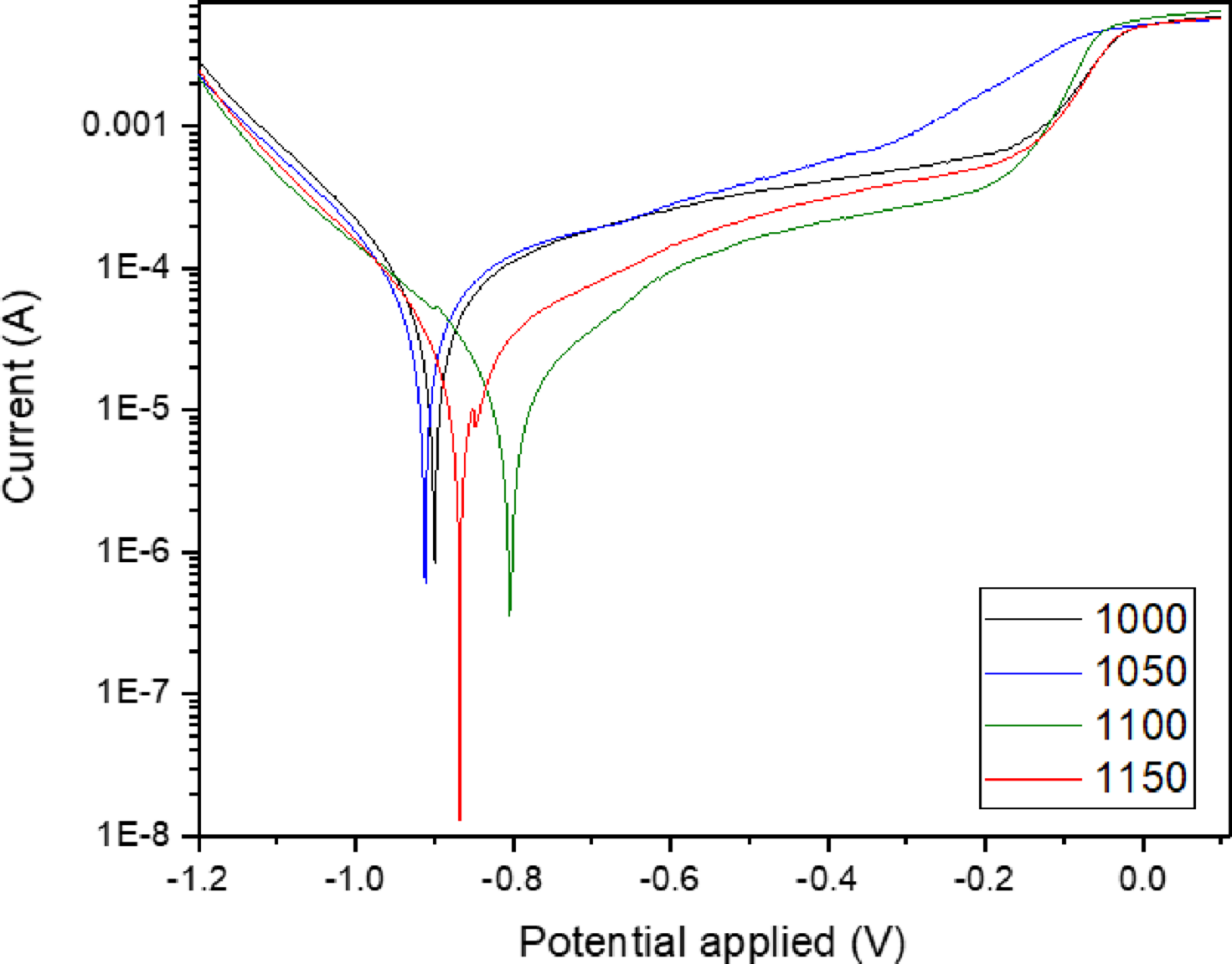
Potentiodynamic polarization curves of HFIHS-processed 316 L stainless steel specimens sintered at 1000–1150 °C after 72 h immersion in 3.5 wt% NaCl at room temperature.

Potentiodynamic polarization tests were conducted after 72 h of immersion in 3.5% NaCl solution to further evaluate corrosion behavior. The polarization curves are shown in Fig. 12, and the extracted electrochemical parameters obtained using Tafel extrapolation are listed in Table 4.

**Fig. 13:**
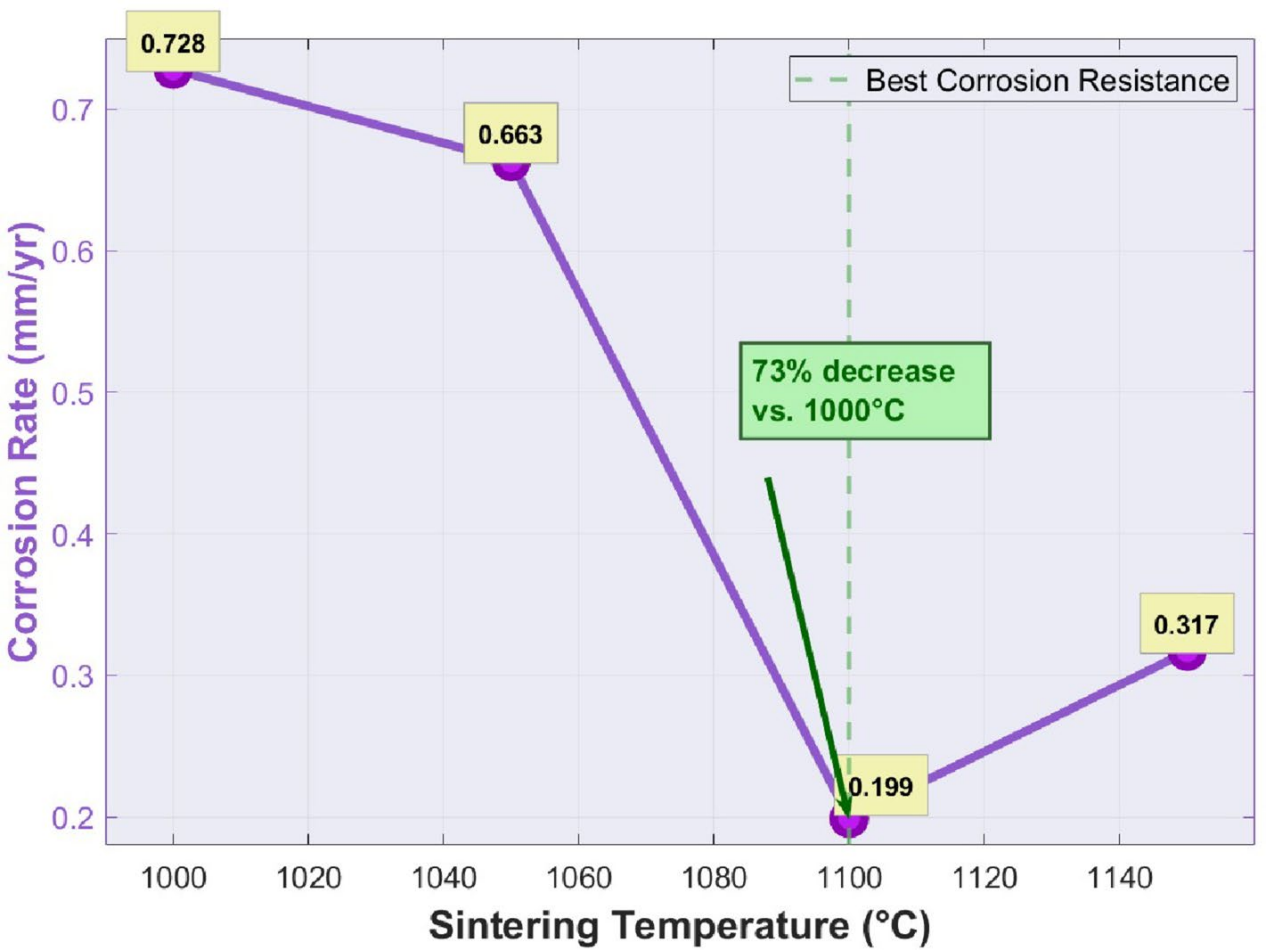
Corrosion rate as a function of sintering temperature for HFIHS-processed 316 L stainless steel after 72 h of exposure to 3.5% NaCl solution.

The polarization data in Table 4; Fig. 13 are consistent with the EIS trends. The corrosion rate reached its minimum at 1100 °C: Jcorr decreased from 6.27 × 10⁻⁵ A cm⁻² at 1000 °C to 1.72 × 10⁻⁵ A cm⁻² at 1100 °C, and the calculated corrosion rate decreased from 0.728 to 0.199 mm year⁻¹, corresponding to a reduction of approximately 73%.

The slight increase in corrosion rate observed for the specimen sintered at 1150 °C compared with the 1100 °C specimen indicates that further densification was not accompanied by a further improvement in the polarization response. Although the relative density increased slightly from 97.27% to 98.61%, grain size also increased at the higher sintering temperature. The observed difference may be associated with microstructural changes accompanying grain coarsening; however, their effects on passive-film development and repassivation were not measured directly. Consequently, the dense microstructure at 1150 °C is consistent with reduced electrolyte-access pathways and a cleaner selected post-corrosion field, whereas the lower corrosion current density and higher polarization resistance measured at 1100 °C indicate a more favorable electrochemical response under anodic polarization. These results suggest that factors in addition to porosity may contribute to the measured corrosion behavior.

Nevertheless, the corrosion rate at 1150 °C remains 56% lower than that of the 1000 °C sample, confirming the substantial benefit of high-temperature HFIHS processing for corrosion resistance enhancement.

Polarization resistance (Rp) follows a similar trend, peaking at 3002.9 Ω for the 1100 °C sample, representing a 249% improvement over the 1000 °C sample (860.13 Ω). The higher Rp is consistent with more limited charge transfer and a more protective electrochemical response under the test conditions; it does not, however, directly demonstrate the formation or stability of a particular passive layer.

**Table 4:** Parameters obtained from Tafel extrapolation of polarization curves after 72 h of exposure.

Sintering Temp (°C)	Ba (V/dec)	Bc (V/dec)	Ecorr (V)	Jcorr (A/cm²)	Corrosion Rate (mm/yr)	Rp (Ω)
1000	0.184	0.3805	−0.896	6.27E-05	0.728	860.13
1050	0.178	0.321	−0.909	5.70E-05	0.663	871.78
1100	0.213	0.268	−0.798	1.72E-05	0.199	3002.9
1150	0.177	0.368	−0.865	2.73E-05	0.317	1907.4

The corrosion current density (Jcorr) obtained from Tafel extrapolation provides a quantitative measure of corrosion rate. As shown in Fig. 12, Jcorr decreases as sintering temperature increases from 1000 °C to 1100 °C, indicating a progressive reduction in corrosion rate. It is important to note that the absolute corrosion rates reported here (0.199–0.728 mm/yr) are higher than those typically reported for wrought or additively manufactured 316 L stainless steel (< 0.05 mm/yr). This difference is expected and is primarily attributable to the residual porosity inherent in sintered powder metallurgy components, which creates occluded micro-environments that concentrate chloride ions and promote localized corrosion. As sintering temperature increases and porosity decreases, the corrosion rate approaches values more consistent with dense 316 L. This behavior can be attributed to improved densification and reduced porosity at higher sintering temperatures, which results in fewer pathways for electrolyte penetration and reduced localized corrosion susceptibility^4,25^.

Within the investigated conditions, the electrochemical results indicate that increasing the sintering temperature from 1000 to 1100 °C improved corrosion resistance. This improvement was associated with increased densification and reduced connected porosity, which can restrict electrolyte-access pathways. The difference between the 1100 and 1150 °C responses suggests that factors beyond densification, potentially including passive-film characteristics, may also contribute; these factors were not measured directly.

### Post-corrosion surface morphology and local EDS analysis

**Fig. 14:**
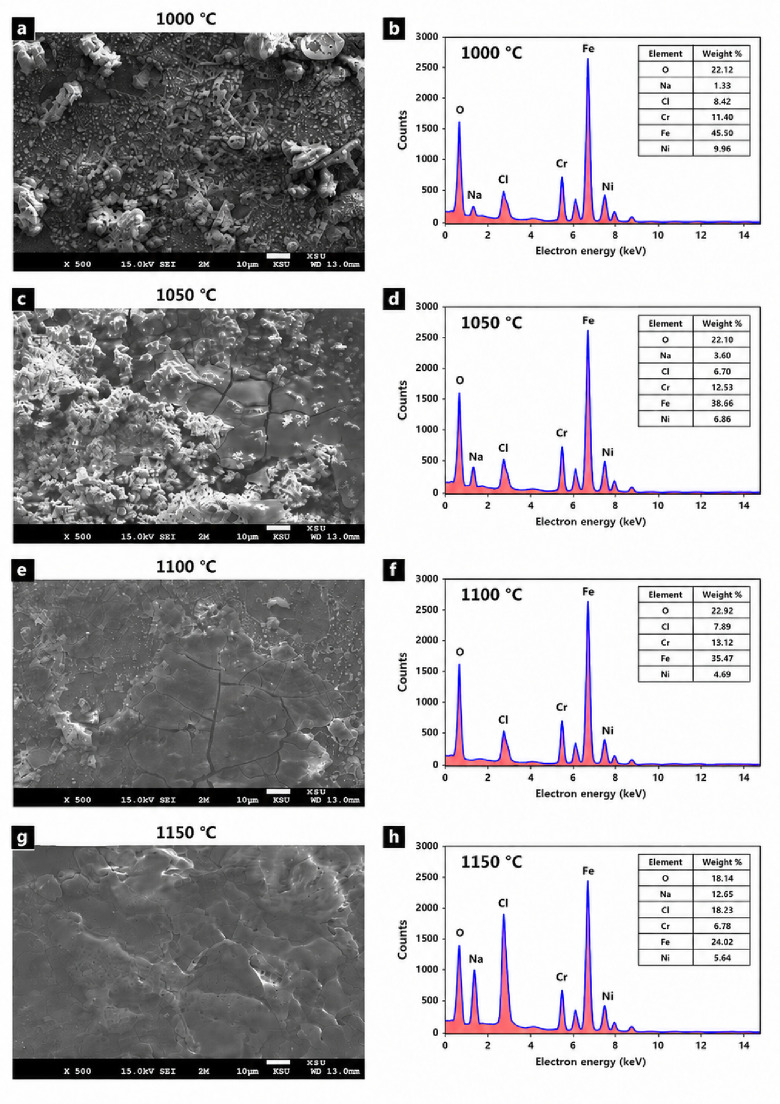
Representative secondary-electron SEM images and corresponding local semi-quantitative EDS spectra of the post-corrosion surfaces of HFIHS-processed 316 L stainless steel specimens sintered at 1000, 1050, 1100, and 1150 °C after 72 h exposure to 3.5 wt% NaCl.

The SEM analysis following electrochemical tests revealed the surface damage which corrosion caused to the sample materials. The post-corrosion SEM micrographs and corresponding EDS analyses are presented in Fig. 14, illustrating the effect of sintering temperature on surface morphology and corrosion behavior.

The SEM images show that the microstructure directly affects the way the material corrodes. The 1000 °C sintered sample shows complete surface corrosion through deep pitting which has formed a dense corrosion product layer that completely covers the entire surface. The white deposits visible in the micrograph correspond to corrosion products formed during the electrochemical reactions in the chloride-containing environment. The deposits display severe corrosion damage because their tight packing arrangement with their uneven surface topography. This is consistent with the high porosity of this sample and its poor performance in the electrochemical tests, where it exhibited the highest corrosion current density and lowest polarization resistance.

The sample sintered at 1050 °C displays a corrosion pattern which resembles the previous sample but with reduced intensity. The surface area continues to show high levels of corrosion products but the substrate base shows better protection in particular sections. The pitting density shows a slight decrease when compared to the 1000 °C sample because this sample showed only a small enhancement in its electrochemical test results.

The minimum polarization-derived corrosion rate occurred at 1100 °C and was approximately 73% lower than that at 1000 °C. The 1150 °C specimen had the highest density and a comparatively clean selected post-corrosion field, confirming that electrochemical performance was governed by more than density alone.

The 1150 °C sample shows a very clean and smooth surface. The surface shows uniform characteristics because it lacks any signs which would indicate specific areas of damage like the samples prepared at lower temperature. Only a few small, isolated regions show slight discoloration and deposits.

The corrosion-product-related elements O, Na, and Cl were detected with different local intensities. Oxygen is consistent with oxygen-containing surface products, whereas Na and Cl are reasonably associated with retained NaCl-derived residues or chloride-containing surface deposits. Their variation among the selected regions supports the heterogeneous surface coverage observed in the SEM images; however, the limited number of local analyses does not permit statistical comparison among the sintering conditions.

Fe remained the principal detected alloying element, with Cr and Ni also present in all selected spectra. Differences in the Fe, Cr, and Ni signals are influenced by local deposit coverage, substrate exposure, and the EDS interaction volume and therefore do not indicate changes in the bulk alloy composition. The more extensive deposits visible at 1000 and 1050 °C are consistent with their less favorable electrochemical response, whereas the selected 1100 and 1150 °C fields appear comparatively less covered. Nevertheless, the visually cleaner 1150 °C field does not override the polarization results, which identified the 1100 °C specimen as the most favorable electrochemical condition.

It should also be noted that EIS characterizes the electrochemical impedance of the interface under small-perturbation conditions, whereas post-corrosion surface morphology reflects the visible extent of surface damage in selected fields after exposure. Consequently, the specimen with the cleanest selected post-corrosion field need not exhibit the lowest polarization current. In the present study, the highly compact microstructure at 1150 °C is consistent with restricted electrolyte-access pathways, whereas the lower corrosion current density and higher polarization resistance at 1100 °C indicate a slightly more favorable electrochemical response. These measurements suggest, but do not directly establish, differences in passive-film characteristics.

### Correlation between microstructure and corrosion behavior

The combined microstructural and electrochemical results show an association between densification, pore morphology, and corrosion behavior. Increasing sintering temperature reduced pore-area fraction, mean pore size, and the two-dimensional connectivity, while the corrosion response improved markedly from 1000 to 1100 °C. These observations support a microstructure-dependent interpretation but do not establish a single causal mechanism.

The quantitative results in Figs. 8, 9 and 10 show that the lower-temperature specimens contained larger and more connected pores. Such connected porosity can provide electrolyte-access pathways and promote occluded chloride-rich environments. This interpretation is consistent with the more extensive deposits observed on the selected 1000 and 1050 °C post-corrosion surfaces and their less favorable electrochemical response.

At 1100 and 1150 °C, the residual pores were smaller, more circular, and less connected. This microstructural change is expected to reduce continuous electrolyte pathways and localized occluded sites. The improved EIS and polarization response is consistent with this reduced connectivity, although passive-film chemistry and local surface condition were not measured directly. The SEM images in Fig. 7 show increasing grain size and improved consolidation with temperature. However, top-view SEM images alone cannot demonstrate defect-free grain boundaries, exclude intergranular attack, or establish passive-film uniformity. Accordingly, the corrosion interpretation is based primarily on the electrochemical measurements and quantitative pore analysis, with SEM used as supporting morphological evidence.

Although the specimen sintered at 1150 °C exhibited the highest relative density (98.61%) and the lowest porosity (1.39%), resulting in the cleanest post-corrosion surface morphology (Fig. 14), this should not be interpreted as indicating the highest electrochemical corrosion resistance. Surface morphology reflects visible damage in selected fields after exposure, whereas potentiodynamic polarization measures the electrochemical response during the scan. The 1100 °C specimen exhibited the lowest corrosion current density and the highest polarization resistance, indicating the most favorable polarization response among the tested conditions. This result is consistent with, but does not directly establish, differences in passive-film behavior. The slightly higher corrosion current at 1150 °C occurred concurrently with a marginal increase in density and pronounced grain coarsening. Grain coarsening may therefore be associated with the change in corrosion response, but a causal relationship cannot be established without direct passive-film chemistry and cross-sectional corrosion-penetration analysis. Accordingly, 1100 °C was the electrochemical optimum under the present test conditions, whereas 1150 °C maximized densification and exhibited the cleanest selected post-corrosion field. Overall, the results suggest that density alone does not account for the observed corrosion response.

Overall, direct HFIHS produced increasingly dense microstructures as the sintering temperature increased. Reduced porosity and pore connectivity were associated with improved corrosion performance from 1000 to 1100 °C. The difference between the 1100 and 1150 °C responses may be associated with passive-film behavior, grain coarsening, or other surface-related factors, but the present measurements do not resolve their individual contributions. Additional surface-chemistry and cross-sectional measurements are required to test these interpretations.

### Declarations of limitations

While this study demonstrates that High-Frequency Induction Heat Sintering (HFIHS) significantly reduces the required pressure and processing time to achieve high-density 316 L stainless steel, few limitations must be acknowledged. First, the use of a graphite die under vacuum at temperatures up to 1150 ∘C presents a potential risk of carbon diffusion into the 316 L matrix, which may affect localized sensitization; future work will systematically quantify carbon content and evaluate intergranular corrosion susceptibility. Second, mechanical characterization was restricted to hardness and compression testing; tensile strength, impact toughness, and fatigue performance under cyclic loading must be investigated to fully qualify this material for structural applications. Third, while excellent corrosion resistance was observed in 3.5% NaCl, evaluations in highly acidic media and long-term exposure testing are required. Finally, the scalability of the HFIHS process to complex, non-cylindrical geometries remains constrained by the uniaxial pressing setup.

## Conclusions

This study established the processing–microstructure–property relationships of gas-atomized 316 L stainless steel consolidated by direct pressure-assisted HFIHS at 40 MPa, approximately 50 kHz, 1000–1150 °C, a 5 min hold, and approximately 1 × 10⁻³ Torr. The principal conclusions are summarized below.

1. Increasing the sintering temperature from 1000 to 1150 °C raised the relative density from 88.87% to 98.61% and reduced density-derived porosity from 11.13% to 1.39%, with the most pronounced densification occurring between 1050 and 1100 °C. Fiji-based image analysis independently confirmed this evolution through reductions in pore-area fraction, equivalent pore diameter, and pore connectivity, together with increased pore circularity.
2. The mean grain size increased from 16.48 ± 3.40 μm at 1000 °C to 61.90 ± 13.93 μm at 1150 °C, indicating that microstructural evolution shifted toward grain-growth-dominated behavior after most densification had occurred. Nevertheless, the benefits of pore elimination and stronger interparticle bonding outweighed the effect of grain coarsening: mean Vickers hardness increased from 156.5 ± 5.2 to 263.0 ± 14.4 HV10, and the condition-level maximum compressive stress increased from 284 to 379 MPa.
3. Corrosion performance did not increase monotonically with density. The lowest polarization-derived corrosion rate was obtained at 1100 °C (0.199 mm year⁻¹), approximately 73% below that measured at 1000 °C, whereas the 1150 °C specimen exhibited the highest density and the least visible damage in the selected post-corrosion field. This distinction identifies 1100 °C as the electrochemical optimum and 1150 °C as the densification and mechanical-property optimum within the investigated range, suggesting that density alone did not determine the measured corrosion response.
4. At 1150 °C, direct HFIHS achieved 98.61% relative density at 40 MPa with a 5 min hold, compared with 94.62% for the process-specific conventional press-and-sinter baseline after compaction at 1600 MPa. The results therefore demonstrate the potential of direct HFIHS for rapid, low-pressure consolidation of 316 L stainless steel, while not establishing general superiority over SPS/FAST or other consolidation routes.

## Data Availability

The data supporting the findings of this study are available from the corresponding author upon reasonable request.
